# Digestibility of Insect Meals for Nile Tilapia Fingerlings

**DOI:** 10.3390/ani9040181

**Published:** 2019-04-20

**Authors:** Táfanie Valácio Fontes, Kátia Rodrigues Batista de Oliveira, Izabella Luiza Gomes Almeida, Tamira Maria Orlando, Paulo Borges Rodrigues, Diego Vicente da Costa, Priscila Vieira e Rosa

**Affiliations:** 1 Department of Animal Science, Universidade Federal de Lavras, 3037 Lavras, MG, Brazil; katiarbo@gmail.com (K.R.B.d.O.); izabellaluizaga@outlook.com (I.L.G.A.); tamira_maria@yahoo.com.br (T.M.O.); pborges@dzo.ufla.br (P.B.R.); priscila@dzo.ufla.br (P.V.R.); 2 Department Institute of Agricultural Sciences, Universidade Federal de Minas Gerais, 135, Montes Claros, MG, Brazil; diego2@ufmg.br

**Keywords:** aquaculture, beetle, cockroach, cricket, *Oreochromis niloticus*, sustainability

## Abstract

**Simple Summary:**

Insects can be a source of nutrients for aquatic organisms, replacing scarce or unsustainable foods. The diversity of insect species contributes to their variable nutritional composition, which fish may digest differently. Nile tilapia is a very important fish for aquaculture, which requires great quantities of quality protein and other dietary nutrients on its initial rearing phase. Therefore, it is important to better understand the technical feasibility of using insect meal as a nutrient and energy source for Nile tilapia fingerlings. In this study, *Tenebrio molitor* larvae meal showed the highest apparent digestibility coefficient, being attested as a potential alternative feed for Nile tilapia fingerlings. Those findings may contribute to sustainable development of the tilapia production around the world.

**Abstract:**

Insects are a valuable source of nutrients for fish, but little is known about their nutritional value for Nile tilapia fingerlings. To evaluate the nutritional value and energy apparent digestibility coefficient (ADC) of five insects for Nile Tilapia male fingerlings, 900 fish were distributed in 18 fiberglass conic tanks, in a completely randomized design, with six dietary treatments (control, *Nauphoeta cinerea* meal (NCM) (Blattodea), *Zophobas morio* larvae meal (ZMM) (Coleptera), *Gromphadorhina portentosa* meal (GPM) (Blattodea), *Gryllus assimilis* meal (GAM) (Orthoptera) and *Tenebrio molitor* larvae meal (TMM) (Coleptera)) and three replicates (tanks), each containing 50 fish. The control diet had no insect meal included and the other five treatments comprised 80% commercial diet and 20% test ingredient with 0.1% chromic oxide as an inert marker. TMM presented a higher ADC for dry matter, protein, corrected protein and chitin than to other treatments (*p* < 0.01). GPM presented the highest ADC for lipids (*p* < 0.01). In general, the TMM presented better ADC of nutrients and energy and all the insect meals evaluated are potential feed for Nile tilapia fingerlings.

## 1. Introduction

The use of alternative ingredients in aquaculture feeds is intended to minimize the dependence on scarce, expensive or unsustainable feedstuff. The main ingredients traditionally used in fish feed are commodities whose price is set by the inconstant global supply and demand. Soybean meal is one of the most used vegetal protein sources in aquafeed formulation due to its high protein content, and amino acid profile as well as its price [1]. However, soybean meal can cause loss of intestinal integrity in fish, leading to non-absorption of nutrients, and impairing fish growth [2]. Another valuable ingredient used in aquaculture feed is fishmeal, but its high global demand has led to overfishing, natural fish stock reduction and price fluctuations [3]. Competition with other sectors, such as pet food, for this feedstuff is increasing and may result in a supply shortage and high prices [3]. Therefore, studies on alternative feeds for aquatic organisms are very important.

Insect meal has stood out as an alternative ingredient to be included in animal feeds [4]. Insects are a natural food source for marine and freshwater fish species, including Nile tilapia [5,6,7]. In general, insects have high protein content, essential amino acids, lipids, minerals and vitamins [8,9] and their nutritional composition may vary based on species, life stage and rearing conditions [8,9].

Studies in recent years have evaluated the use of insects as feed for freshwater fish, such as African catfish *Clarias gariepinus* [10,11], rainbow trout *Oncorhynchus mykiss* [12], Jian carp *Cyprinus carpio* [13], yellow catfish *Pelteobagrus fulvidraco* [14], red tilapia *Oreochromis sp*. [15] and Nile tilapia *Oreochromis niloticus* [16]. Freccia et al. (2016) [16] did not observed differences on growth performance and hematological parameters of Nile Tilapia fingerlings fed 0, 5%, 10%, 15% or 20% cinerea cockroach *Nauphoeta cinerea* meal (NCM). The authors concluded that cinerea cockroach meal may be used as feed for Nile tilapia fingerlings. Jabir et al. (2012) [15] compared the apparent digestibility coefficient (ADC) of *Zophobas morio* larvae meal (SMM) and fishmeal (FM), the main dietary protein sources for juvenile red tilapia. Protein and lipid ADC in the SWM based diet was lower (50.53% ± 6.08% and 69.76% ± 3.72%, respectively) than in the FM diet (77.48% ± 0.53% and 91.51% ± 0.21%, respectively). The authors concluded that the ADC of SWM would need to be improved for it to be a reasonable alternative for a partial or complete replacement of FM in the red tilapia diet. 

Nile tilapia is the fourth most farmed fish species around the world, representing 8% of total global inland production [17] and 36% of the total Brazilian fish farming [18]. Nile tilapia is an omnivorous fish with some advantages on chitin degradation, because polysaccharides are part of the composition zooplanktons, a natural feed for tilapia [19]. The ability to degrade chitin from insect and shrimp meal through the presence of chitinolytic enzymes from pancreatic and gastric mucosa has already been clarified for some teleost fish species [20,21,22]. Considering tilapia´s possible advantages in chitin degradation and its omnivorous feeding habits [23], insect meal are expected to become a feasible feed for Nile tilapia fingerlings. As the ADC provides information about how much a fish has digested and retained a feed, knowing the digestibility of novel dietary ingredients is the basis to assess their bioavailability and hence their sustainability for inclusion in fish diets.

Therefore, the aim of this study was to assess the nutrient and energy apparent digestibility coefficient from five insect meals; two cockroach species (*Nauphoeta cinerea* and *Gromphadorhina portentosa*), one cricket species (*Gryllus assimilis*) and two species of tenebrio (*Zophobas morio* and *Tenebrio molitor*) for Nile tilapia fingerlings. 

## 2. Materials and Methods 

### 2.1. Animals and Experimental Design

All procedures involving fish in this study were assessed and approved by the Animal Ethics Committee from the Federal University of Minas Gerais (protocol number 107/2017). The committee only assess studies involving animals from the phylum Chordata, subphylum Vertebrata, which is why the procedures with involving insects are not involved in the mentioned protocol. 

An in vivo digestibility trial was performed to assess the apparent digestibility coefficients (ADC) of the dietary nutrients and energy. The feeding trial was carried out at the Experimental Aquaculture Station of the Animal Science Department at the Federal University of Lavras in a completely randomized design with six dietary treatments (one control with no insect meal and five insect meals) and three replicates (fiberglass cylindro-conic tanks with 250 L capacity) each containing 50 reversed male Nile Tilapia fingerlings *Oreochromis niloticus* (3.0 ± 0.2 g of mean weight). The trial was set up in a recirculating system adapted to the Guelph system, equipped with a fiber mesh pad filter, a submerged biological filter and an ultraviolet filter. The system was supplied with constant aeration, and thermoregulation (26 ± 1 °C). Oxygen saturation in the water was 85% ± 2%; the ammonia and nitrite concentration amounted to 0.2 ± 0.1 and 0.3 ± 0.1 mg/L, respectively. A 12-h light/dark cycle was adopted. 

### 2.2. Diets, and Feeding Trial

The insect meals were obtained from the Laboratory of Entomoculture of at the Institute of Agricultural Sciences of the Federal University of Minas Gerais (Montes Claros, MG, Brazil). All the insects were reared on a vegetal diet (soybean, corn and wheat), killed by immersion in boiling water, dried in a forced air oven (50 °C for 48 h) and milled in an electric screw meat grinder (Botini 1/3cv, Brazil). *Nauphoeta cinerea*, *Gromphadorhina portentosa* and *Gryllus assimilis* were harvested as adults while *Zophobas morio* and *Tenebrio molitor* were harvested as larvae.

The fish diets comprised 20% test ingredient (insect meals) and 80% control diet (commercial diet with 32% crude protein, Linha aquos Tropical––TOTAL) with 0.1% chromium oxide (Cr_2_O_3_) as an inert marker. The control diet was used as the reference diet, and the data collected from this group was used to calculate the test ingredient’s ADCs, as described below. After 10 days of adaptation to laboratory conditions, the fish were fed three times daily to the point of apparent satiation for 15 days with the experimental diets, with an average of 18 g of feed per feeding per tank. After the last daily feeding, tanks were cleaned and plastic tubes connected at the bottom of each tank for feces collection overnight, for 15 days. The next morning, the tubes were removed and feces dried in a forced-air drying oven (60 °C) for 48 h and stored in a freezer (−20 °C) for laboratory analysis.

### 2.3. Chemical Composition and Digestibility Analysis

Chemical analyses of the test ingredients, diets and feces were conducted according to Association of Official Agricultural Chemists (AOAC) [24] for dry matter (930.15), crude protein (968.06) and ash (942.05). The chemical composition of the insect meals and diets are shown in Table 1 and Table 2, respectively. Corrected crude protein was determined by the Kjeldahl method; however, the nitrogen-to-protein correction factor used was 4.76 (Kp = 4.76) instead of the usual 6.25, as described by Janssen et al. [25], in which nitrogen from chitin are not considered. Crude lipid was quantified following Folch’s [26] methodology and chitin according to Souto’s [27]. Gross energy was measured in a calorimetric bomb (IKA C7000, Staufen, Germany) and chromium oxide following Bremer Neto et al. [28]. 

The ADC of nutrients and energy from the experimental diets was calculated following the equations Bureau and Hua [29] proposed. 

ADC (1) and ADCi (2) were calculated according to the following equations:

(1) ADC (%)=100−[100×(Id/If×Nf/Nd)] (1),

(2) ADCi (%)=ADCdt+[ADCdt−ADCref)×(r×Nref/i×Ni)] (2),

in which

(1) *ADC* is the apparent digestibility coefficient;
  *Id* is chromic oxide’s concentration in the diet (%);
  *If* is chromic oxide’s concentration in the feces (%);
  *Nd* is the nutrient’s concentration in the diet (%);
  *Nf* is the nutrient’s concentration in the feces (%);
(2) *ADCi* is the nutrient’s apparent digestibility coefficient in the test ingredient;
  *ADCdt* is the nutrient’s apparent digestibility coefficient in the test diet;
  *ADCref* is the nutrient’s apparent digestibility coefficient in the reference diet;
  *r* is the reference diet’s proportion in the test diet (0.6994);
  *i* is the test ingredient’s proportion in the test diet (0.3);
  *Nref* is the nutrient’s concentration in the reference diet (% as fed);
  *Ni* is the nutrient’s concentration in the ingredient (% as fed).

### 2.4. Statistical Analyses

Data is expressed as mean and standard error of the means (SEM). Normality and homogeneity of variances were tested using the Shapiro–Wilk and Levene tests, respectively. Data were analyzed by one-way ANOVA followed by Tukey’s multiple range test at a 1% probability level. All statistical analyses were performed using SPSS 22.0 software package (IBM, Chicago, IL, USA).

## 3. Results

The ADC of the insect meal’s dry matter, protein, corrected protein, energy, lipids and chitin are presented in Table 3. Significant differences between treatments were observed for all the parameters evaluated (*p* < 0.01). *Tenebrio molitor* meal presented a higher ADC for dry matter, protein, corrected protein and chitin than the other tested ingredients (*p* < 0.01). The cricket *Gryllus* *assimilis* meal had the lowest digestibility for protein and corrected protein (*p* < 0.01). For energy, *Zophobas morio* and *T molitor* meals presented higher ADC’s than the other treatments (*p* < 0.01), and *G. assimilis* and the cockroaches, *Nauphoeta* *cinerea* and *Gromphadorhina* *portentosa,* did not differ. The lipid ADC of *G. portentosa* meal was the highest among the treatments (*p* < 0.01). The highest chitin ADC was found in *T. molitor* meal and the lowest in *N. cinerea* meal (*p* < 0.01). 

## 4. Discussion

Insects have been widely studied as fish feed in the last few years, but only a few studies have focused on the nutrient and energy digestibility. The variation in nutrient and energy digestibility is based on the insect species, life stage, and the fish species [4], and may not be considered the only aspect to make recommendations regarding their inclusion in fish diets, mainly when talking about insect meal. Authors have reported that the inclusion of insect meals in fish diets is possible without damaging growth performance [30,31]. However, it seems to depend on the inclusion level, fish species, insect species and nutrient composition. In the present study, it was possible to observe that different insect meals presented distinguished chemical compositions and nutrients and energy ADCs, so their inclusion in practical aquafeeds formulations need to be suitable regarding those specific characteristics, to maintain the quality and supply the nutritional requirements. 

A reduction in nutrient digestibility due to the presence of chitin was expected [4,12], as Piccolo et al. reported [30] for gilthead sea bream juveniles fed with increasing dietary levels of *T. molitor* meal. 

Low values of dry matter ADC are found for *N. cinerea*, *G. portentosa* and *G. assimilis*, ranging from 42.6% to 61.7%. This decrease in dry matter ADC can be explained by the feed’s bromatological composition, in which higher chitin values were found. Shiau and Yu [32], using diets with chitin levels of 0%, 2%, 5% and 10%, found that the highest level of supplementation was accompanied by a lower level of dry matter ADC for Nile tilapia. We can observe that for *T. molitor* and *Z. morio* the dry matter ADC was higher than the other diets’, which corroborates Shiau and Yu’s [32] research because *T. molitor* and *Z. morio* had the lowest chitin percentage in their composition.

We have shown in the results the corrected protein parameter because chitin is a nitrogen-compound, so Kjeldahl’s method may overestimate the insect meal’s protein content [9]. Nevertheless, even fulfilling the protein correction, the values of chitin ADC found in this study are still considered high [9]. According to Longvah et al. [33] the chitin may interfere with the dietary utilization of protein, where a reduction on protein digestibility due to an increase in the chitin content is expected [12]. *N. cinerea*, *G. portentosa* and *G. assimilis* meals, in the present study, presented lower protein ADC, which can be connected to this feed’s protein quality, that is, their amino acid composition or even because of their higher chitin content. However, tenebrios, *T. molitor,* and *Z. morio* meals showed dry matter ADC close to the reported values for fishmeal and soybean meal [34] for Nile tilapia. The protein ADC of *Tenebrio molitor* meal was also close to that of those feeds [34].

Moreover, the type of chitin matrix in insects may negatively influence chitinase activity and thus protein digestibility [35]. Those enzymes are essential for the breakdown of chitin [36], the main structural polysaccharide present in insect exoskeletons [9]. The efficacy of chitin utilization by monogastric animals is frequently discussed in connection with the presence, or lack of, of chitinolytic enzymes; however, some studies confirmed the presence and activity of chitinolytic enzymes in various organs of fish species, such as the gastric mucosa, intestinal mucosa, pyloric caeca and pancreas [37,38,39,40]. As an omnivorous fish species with a great capacity to feed on plankton, Nile tilapia may have some advantages in chitin degradation, once that polysaccharide is present in zooplankton composition [41]. The feeding nature and significant intake of chitin make it likely that chitinolytic enzymes play an important role on tilapia digestive physiology. Köprücü and Özdemir [34] assessed the digestibility of chitin from crustacean meals (*Gammarus kischineffensis* and *Astacus leptodactylus*) for Nile tilapia and reported chitin ADC of 71.5% and 69.3%, respectively. For cobia *Rachycentron canadum*, chitin ADC from brown shrimp (*Penaeus aztecus)* and crab meals (*Brachyura*) were 78.2% and 66.8% respectively [30]. The chitin ADCs those authors reported were close to those presented in this study, which ranged from 59.8% to 81.3%. Studies on chitinase and chitobiase activity as well as the absorption and utilization capacity of chitin and its derivates, such as N-acetyl glucosamine by Nile tilapia are needed.

According to Tanaka et al. [42], chitin may inhibit the absorption of lipids in the gut, increasing their excretion and, thereby affecting their digestibility coefficient. The insect meal chitin content in this study ranged from 12.01% to 28.94%, however, chitin did not seem to influence lipid digestibility, probably due to its high digestibility. The *G. portentosa* meal presented the highest chitin content (28.94%) and the highest lipid ADC (98.8%) among treatments. Additionally, *N. cinerea* and *G. assimilis* showed 91.6% and 87.9%, respectively, and this reduction was not a reflex of chitin levels, as Shiau and Yu [32] demonstrated, but can it be attributed to the insect’s fatty acid quality; therefore this should be investigated.

Nandeesha et al. [43] reported that insect oil from silkworm pupa (*Bombyx mori*) have high amounts of digestible fatty acids and were as effective as sardine oil in providing energy to induce fast growth in common carp (*Cyprinus carpio*) fed 3%, 6% and 9% silkworm meal or sardine oil. Those authors also reported that fat digestibility increased with an increasing inclusion level of non-defatted insect meal. Lipid digestibility among fish is known to be quite variable and dependent on many factors, such as the inclusion level and fat source [44]. In general, the lipid digestibility found in this study, although it differs among treatments, was relatively high and may be equated with conventional lipid sources such as full fat vegetable meals [44]. 

The *G. portentosa* meal presented the highest percentage of lipid digestibility (98.8%) and low protein and corrected protein digestibility (61.6% and 58.3% respectively). Nandeesha et al. [43] reported that the ADC of protein is low when the fat ADC is high for common carp fed silkworm pupa meal. 

## 5. Conclusions

In general, all the insect meals studied are potential alternative ingredients for aquafeed formulation. *Tenebrio molitor* larvae meal is the most suitable feed for Nile tilapia fingerlings among the insect meals evaluated. Further investigation is needed to understand the nutritional value and optimal inclusion level of various insect meals for fish.

## Figures and Tables

**Table 1:** Centesimal composition analysis of the insect meals (dry matter basis).

Nutrients	*N. cinerea*	*Z. morio*	*G. portentosa*	*G. assimilis*	*T. molitor*
Dry matter (%)	93.69	94.56	94.60	92.41	95.95
Protein (%)	64.78	49.91	69.94	62.09	47.82
Corrected Protein * (%)	39.04	30.11	37.45	39.75	28.85
Energy (MJ Kg^-1^)	30.7	26.8	21.2	24.0	26.6
Lipids (%)	22.68	33.05	12.97	18.14	31.69
Ash (%)	3.83	2.77	4.03	4.48	2.61
Chitin (%)	24.36	22.48	28.94	22.34	12.01

* Corrected crude protein was calculated by applying a nitrogen-to-protein conversion factor of Kp = 4.76 [25].

**Table 2:** Centesimal composition of the experimental diets (dry matter basis).

Nutrients	*N. cinerea*	*Z. morio*	*G. portentosa*	*G. assimilis*	*T. molitor*	Control
Dry matter (%)	90.99	94.08	94.42	95.14	94.77	94.43
Protein (%)	36.29	34.95	32.02	33.55	31.30	34.29
Corrected Protein * (%)	27.50	29.91	24.99	27.32	24.60	34.29
Energy (MJ·Kg^-1^)	19.1	18.6	19.2	19.0	18.1	19.3
Lipids (%)	8.86	9.17	7.13	8.69	9.40	4.79
Ash (%)	11.02	10.79	10.23	10.95	10.27	11.87
Chitin (%)	5.37	4.47	7.69	5.02	3.87	-

* Corrected crude protein was calculated by applying a nitrogen-to-protein conversion factor of Kp = 4.76 [25].

**Table 3:** Apparent digestibility coefficients (%) of the five insect meals evaluated for Nile tilapia fingerlings.

Nutrients	*N. cinerea*	*Z. morio*	*G. portentosa*	*G. assimilis*	*T. molitor*	SEM	*p*-Value
Dry matter	61.7 ^b^	83.2 ^c^	48.2 ^a^	42.6 ^a^	95.8 ^d^	6.0	<0.0001
Protein	69.6 ^c^	70.0 ^c^	61.6 ^b^	39.7 ^a^	85.4 ^d^	4.2	<0.0001
Corrected Protein *	67.7 ^c^	74.3 ^c^	58.3 ^b^	38.9 ^a^	92.4 ^d^	5.2	<0.0001
Energy	58.4 ^a^	80.1 ^b^	47.4 ^a^	47.0 ^a^	82.1 ^b^	4.7	<0.0001
Lipids	91.6 ^ab^	93.5 ^b^	98.8 ^c^	87.9 ^a^	90.6 ^ab^	1.1	<0.0001
Chitin	59.8 ^a^	73.6 ^bc^	69.6 ^b^	76.2 ^c^	81.3 ^d^	2.1	<0.0001

Values presented as means (*n* = 3) and pooled standard error of the mean (SEM). ^a–d^ means in the same row with different superscripts are different at *p* < 0.01. * Corrected crude protein was calculated by applying a nitrogen-to-protein conversion factor of Kp = 4.76 [25].
